# Aggregation of human osteoblasts unlocks self-reliant differentiation and constitutes a microenvironment for 3D-co-cultivation with other bone marrow cells

**DOI:** 10.1038/s41598-024-60986-8

**Published:** 2024-05-06

**Authors:** Sabrina Marozin, Birgit Simon-Nobbe, Astrid Huth, Evelyn Beyerer, Laurenz Weber, Andreas Nüssler, Günter Lepperdinger

**Affiliations:** 1 Department of Biosciences and Medical Biology, University Salzburg, 5020 Salzburg, Austria; 2 Siegfried Weller Institut (SWI) | BG Klinik Tübingen, Tübingen, Germany

**Keywords:** Spheroid, Osteogenesis, Osteocyte, Calcification, Endothelial cells, Mesenchymal stroma cells, Cell biology, Extracellular matrix, Regeneration, Stem-cell differentiation, Stem-cell niche, Mesenchymal stem cells, Bone development

## Abstract

Skeletal bone function relies on both cells and cellular niches, which, when combined, provide guiding cues for the control of differentiation and remodeling processes. Here, we propose an in vitro 3D model based on human fetal osteoblasts, which eases the study of osteocyte commitment in vitro and thus provides a means to examine the influences of biomaterials, substances or cells on the regulation of these processes. Aggregates were formed from human fetal osteoblasts (hFOB1.19) and cultivated under proliferative, adipo- and osteoinductive conditions. When cultivated under osteoinductive conditions, the vitality of the aggregates was compromised, the expression levels of the mineralization-related gene DMP1 and the amount of calcification and matrix deposition were lower, and the growth of the spheroids stalled. However, within spheres under growth conditions without specific supplements, self-organization processes occur, which promote extracellular calcium deposition, and osteocyte-like cells develop. Long-term cultivated hFOB aggregates were free of necrotic areas. Moreover, hFOB aggregates cultivated under standard proliferative conditions supported the co-cultivation of human monocytes, microvascular endothelial cells and stromal cells. Overall, the model presented here comprises a self-organizing and easily accessible 3D osteoblast model for studying bone marrow formation and in vitro remodeling and thus provides a means to test druggable molecular pathways with the potential to promote life-long bone formation and remodeling.

## Introduction

3D in vitro cell culture models exhibit functional morphology in emerging physiological microenvironments. Hence, compared to single cells or cell clusters, microtissues are more likely to guide developmental pathways in a manner comparable to in vivo conditions. Although novel models exist, artificial microtissues can thus provide insights into species-specific processes^1^. In the case of models built from human cells, these are considered unprecedented^2,3^. 3D aggregates also enable long-term experiments. Here, the cells exhibited properties different from those cultivated on plastic in 2D. For instance, major distinctions between 3D-cultivated cells and 2D-cultivated cells include self-organized growth versus contact inhibition in a confluent state, coordinated movements of micromasses versus migration of single cells, or developmental processes governed by tissue-intrinsic parameters versus differentiation triggered by extrinsic cues.

Crowding of cells over longer periods of time provides a firm basis for building a structured extracellular matrix (ECM). This in turn supports the function of cells and decisively promotes fate decisions in the context of dynamic microenvironmental changes^4,5^. In the context of lineage decisions, the presence of only ECM derived from mesenchymal stromal cells can trigger osteogenic differentiation of progenitor cells^6,7^. Additionally, the sizes of cell aggregates are important. The availability of oxygen or nutrients and/or the removal of waste products may become restrictively challenging for cells located in deep layers of micromasses^8^. It has been reported that in many 3D-avascular cell models, necrotic areas emerge within core regions^9,10^. Necrosis is highly indicative of non-physiologic cellular states or unfavorable extracellular conditions.

In vitro models, which may enable the study of osteoblast-osteocyte transition, are of particular interest in the field, as investigations of these cells in experimental animal models or human beings are particularly difficult. We therefore set out to study the cell aggregation of fetal osteoblasts (hFOB1.19) from the perspective of specifying conditions to make cell aggregates appropriate for serving as a substrate and/or providing niches for other cell types commonly found in the bone marrow.

## Results

### hFOB form solid spheroids under proliferative conditions in the absence of a necrotic core

**Figure 1:**
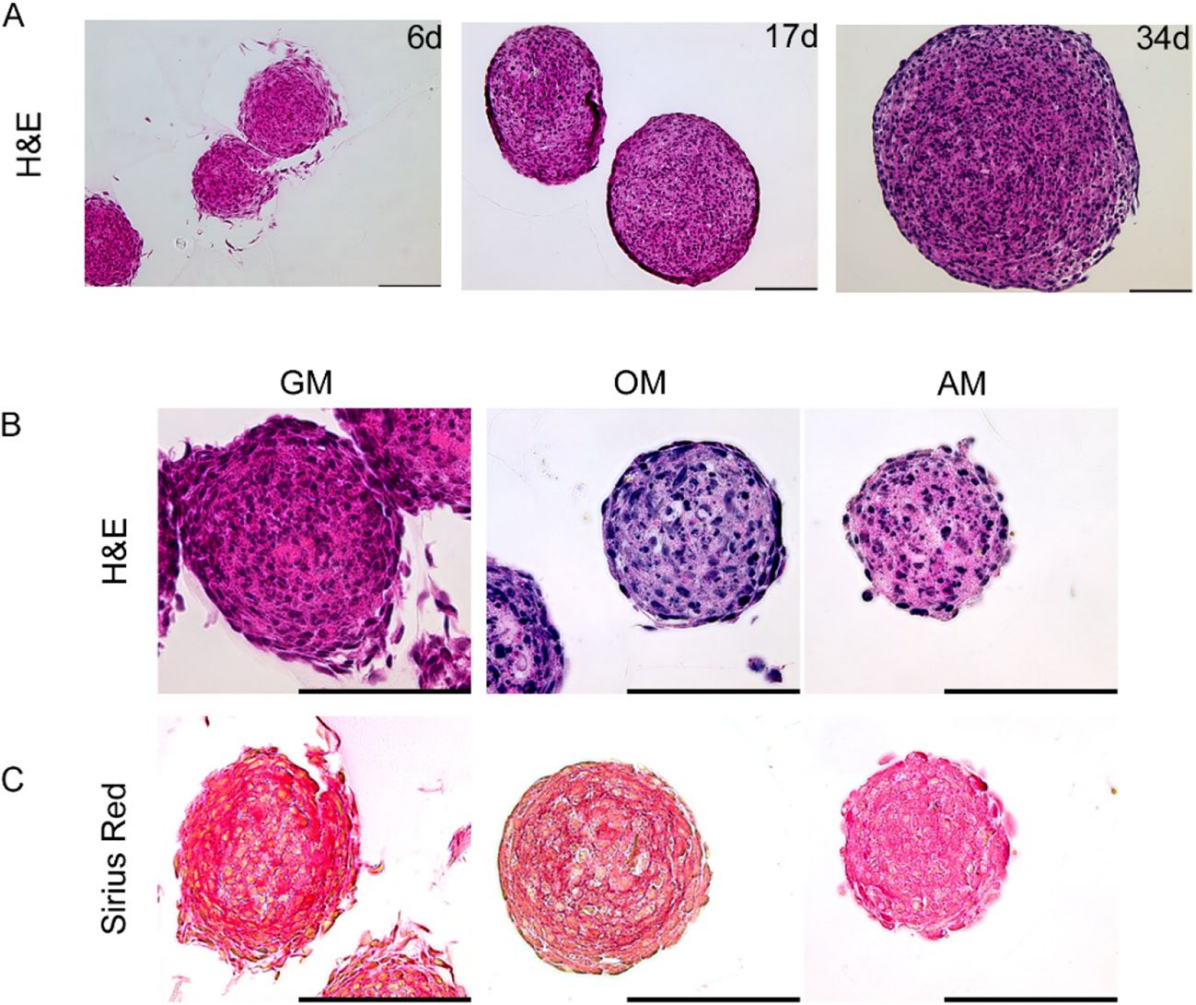
hFOB spheroid development under proliferative, osteo- and adipo-inductive conditions. (**A**) Representative hematoxylin/eosin (H&E) histological images of hFOB cell aggregates grown under proliferative condition (growth medium, GM) over 34 days. Aggregates grown in spheroids over the timespan exhibited no necrotic onset. Scale bar 100 µm. (**B**) Decalcified 6-days-old spheroids grown under proliferative (GM), osteo- (OM) and adipo-inductive (AM) conditions were stained with H&E. Under proliferative condition, spheroids showed a high density of nuclei in the core and no prominent signs of necrosis. In osteo-and adipo-inductive conditions, despite the absence of visible necrosis, more nuclear fragmentation is present. Spheroids in growth medium displayed larger eosinophilic regions. (**C**) Sirius Red staining of spheroids: red stain indicates a high degree of collagen content in spheroids in growth and, to less extent, in osteogenic media. Scale bar 100 µm.

Human fetal osteoblast cells (hFOB 1.19) form spheroids when allowed to aggregate in a non-adherent 3D culture environment. To assess the development of these cell aggregates over time, we first evaluated spheroid appearances under proliferative conditions by performing histological analysis. Spheroids that had grown for more than 30 days showed no apparent signs of necrosis (Fig. 1A). This result was corroborated by the observation that spheroids were generated and maintained under osteogenic or adipo-inductive conditions. Spheroids kept in proliferative conditions continued to grow over time, while those incubated in induction media ceased growth and showed high proportion of fragmented nuclei (Fig. 1B). In addition to exhibiting a rather small diameter, adipogenesis-induced spheroids displayed irregular rough boundaries and an unstructured histological appearance. There was no apparent distinction between cells located in the shell or core areas. Additionally, the osteogenesis-induced spheroids exhibited very little growth over time and were smaller than those preserved in growth medium. Hematoxylin/eosin (H&E) staining of histological sections highlighted a clearly contrasted basophil environment. At any time of culture and culture conditions, the cores exhibited no detectable signs of necrosis. Compared with those in osteogenic and adipogenic media, spheroids in growth media showed strong eosinophilic staining.

Since the extracellular matrix is known to be eosin-positive, sections were also subjected to Sirius red staining for visualization of collagen (Fig. 1C). In this way, structured extracellular matrix organization was observed in spheroids in growth media in all regions, with a particularly high density around the central region. Osteogenic spheroids displayed a ring-shaped, concentric organization of thin layers of matrix between shell cells located in deeper shell layers. In stark contrast, spheroids undergoing adipogenic differentiation showed no characteristic aspects of collagen-specific staining.

### hFOB spheroids display core calcification

**Figure 2:**
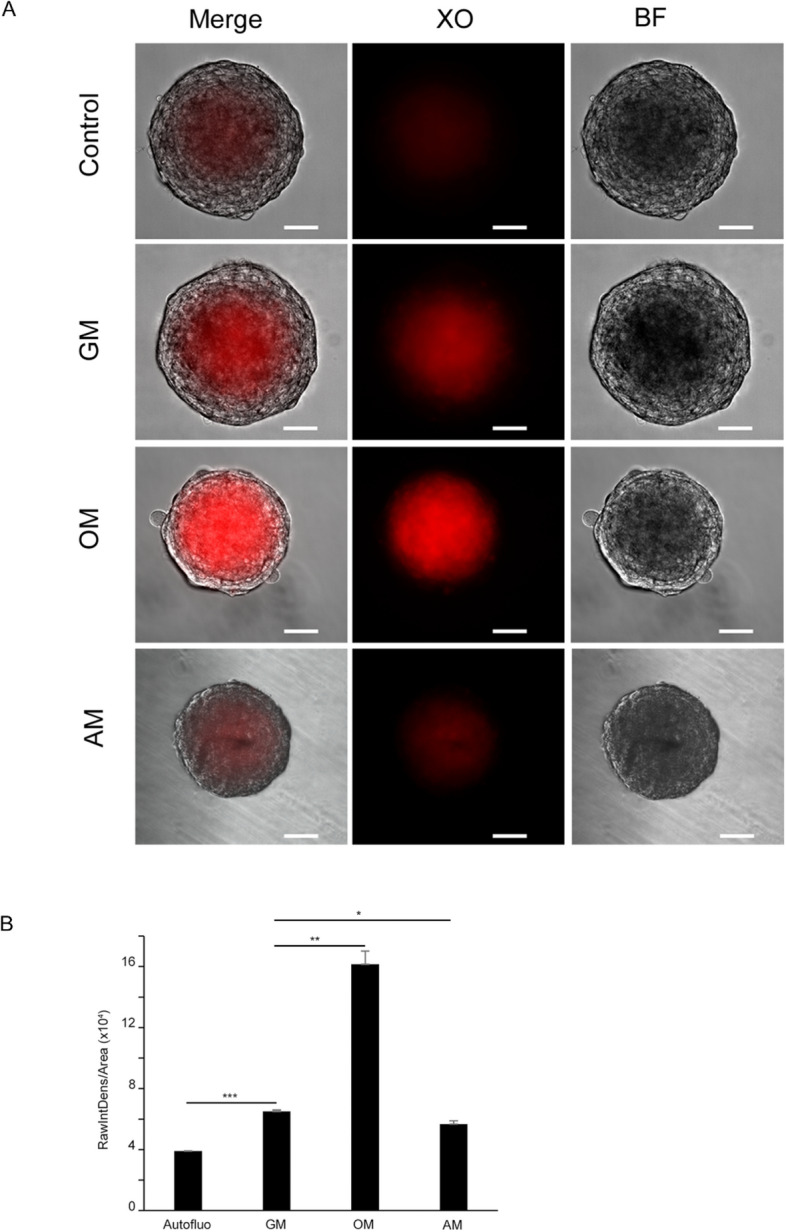
Calcification in the core of hFOB spheroids. (**A**) Microscopic evaluation of core mineralization detected by Xylenol Orange (XO). Representative epifluorescence and brightfield (BF) images of 6-days old spheroids cultivated in the presence of 20 µM XO. Control spheroids were cultured in growth medium in the absence of XO. Spheroids in growth and osteogenic media showed a positive signal for XO (red) in the core, while in adipogenic medium the intensity of the signal was comparable to controls. Scale bar, 50 µm (**B**) Quantitative estimation of XO signal intensity by means of digital image analysis (ImageJ/FIJI). Data represent mean ± s.d of three independent experiments in triplicates. Statistical significance was evaluated by a two-tailed unpaired Student T-test. *P = 0.035; **P = 0.0039; ***P = 0.0006.

Spheroids cultured under the same conditions as described before were stained with Xylenol Orange (XO), which results in red fluorescence when bound to calcium^11^. In comparison to unstained spheroids in growth medium (autofluorescence control), both spheroids in growth medium and those in osteogenic medium displayed a clearly higher fluorescence signal. Additional staining methods applying Calcein Blue and the Von-Kossa-dye method corroborated that extracellular calcium was deposited within the core region merely when keeping spheroids under proliferative conditions (Fig. S1). Furthermore, under comparable culture conditions, accumulating subcellular lipid storage, which is a consistent indication for adipogenic differentiation could not be observed. Notably, PPARγ2 expression was found upregulated upon incubation with adipogenic inducers (Fig. S2). Moreover, after staining with the calcium dye, Xylenol Orange these spheroids were barely distinguishable from unstained controls, hence under these conditions spheroids refrained from depositing calcium in the extracellular spaces of their cores (Fig. 2A,B).

**Figure 3:**
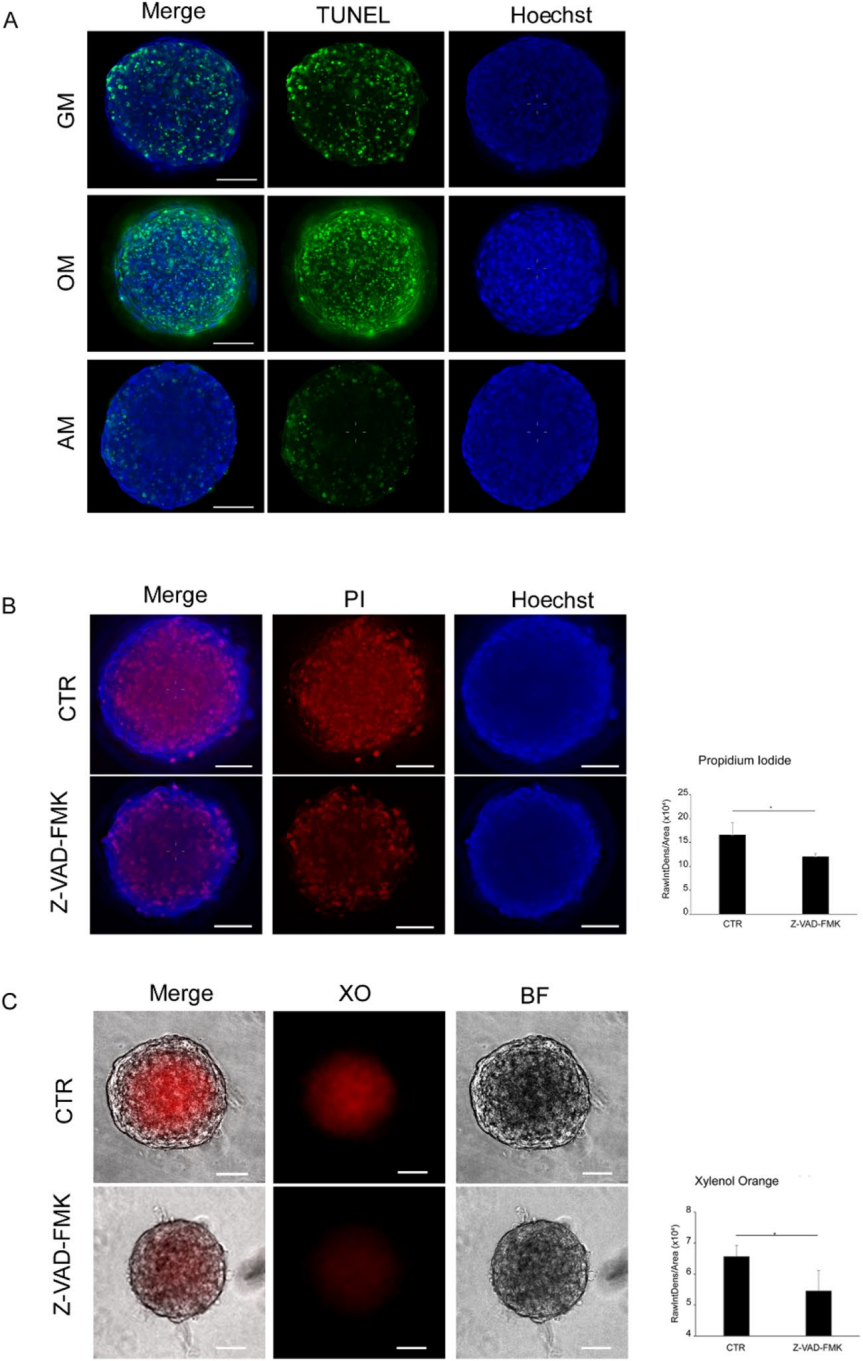
Core calcification and apoptosis. (**A**) TUNEL analysis: representative deconvoluted images of 4-days-old spheroids under different growth conditions containing apoptotic cells (green). Nuclei were counterstained with Hoechst 33342 (blue); scale bar, 50 µM. (**B**) Spheroids were cultured in growth medium (CTR), or in growth medium containing 50 µm of the caspase inhibitor Z-VAD-FMK. Propidium Iodide (PI) and Hoechst 33342 (Hoechst) stain were performed 5 days post-treatment. Scale bar, 50 µm. (**C**) Mineralization was visualized under the same conditions with Xylenol Orange (XO) and brightfield microscopy (BF). Scale bar, 50 µm. Statistical significance was evaluated by a two-tailed unpaired Student T-test. Data represent mean ± s.d of at least two independent experiments in triplicates. * *P* > 0.05.

As reported for various types of large spheroids, only cells in the outermost layer appear to proliferate. We also found expression of Ki-67, a marker for cell proliferation, restricted to the outmost cell layers (Fig. S3). Yet, cells within the deeper region in the core are often prone to die and form characteristic necrotic areas^12^. Therefore, cell death in hFOB spheroids was evaluated applying TdT-mediated dUTP-biotin nick end labeling (TUNEL) analysis (Fig. 3A). Compared with that in the other conditions, the TUNEL green fluorescence signal in the spheroids was strongly increased in the osteogenic medium. Since osteogenic medium appeared to have adverse effects on cell aggregates, we performed further experiments only in growth medium.

As reported elsewhere, caspase inhibition impacts osteogenic differentiation^13,14^, and spheroids that had been maintained under proliferative conditions were treated with 50 µM of the pancaspase inhibitor Z-VAD-FMK. This resulted in fewer Propidium iodide (PI)-positive nuclei (Fig. 3B), and both the spheroid diameter and the XO signal were clearly reduced (Fig. 3C).

### 3D culture supports osteoblast self-differentiation

**Figure 4:**
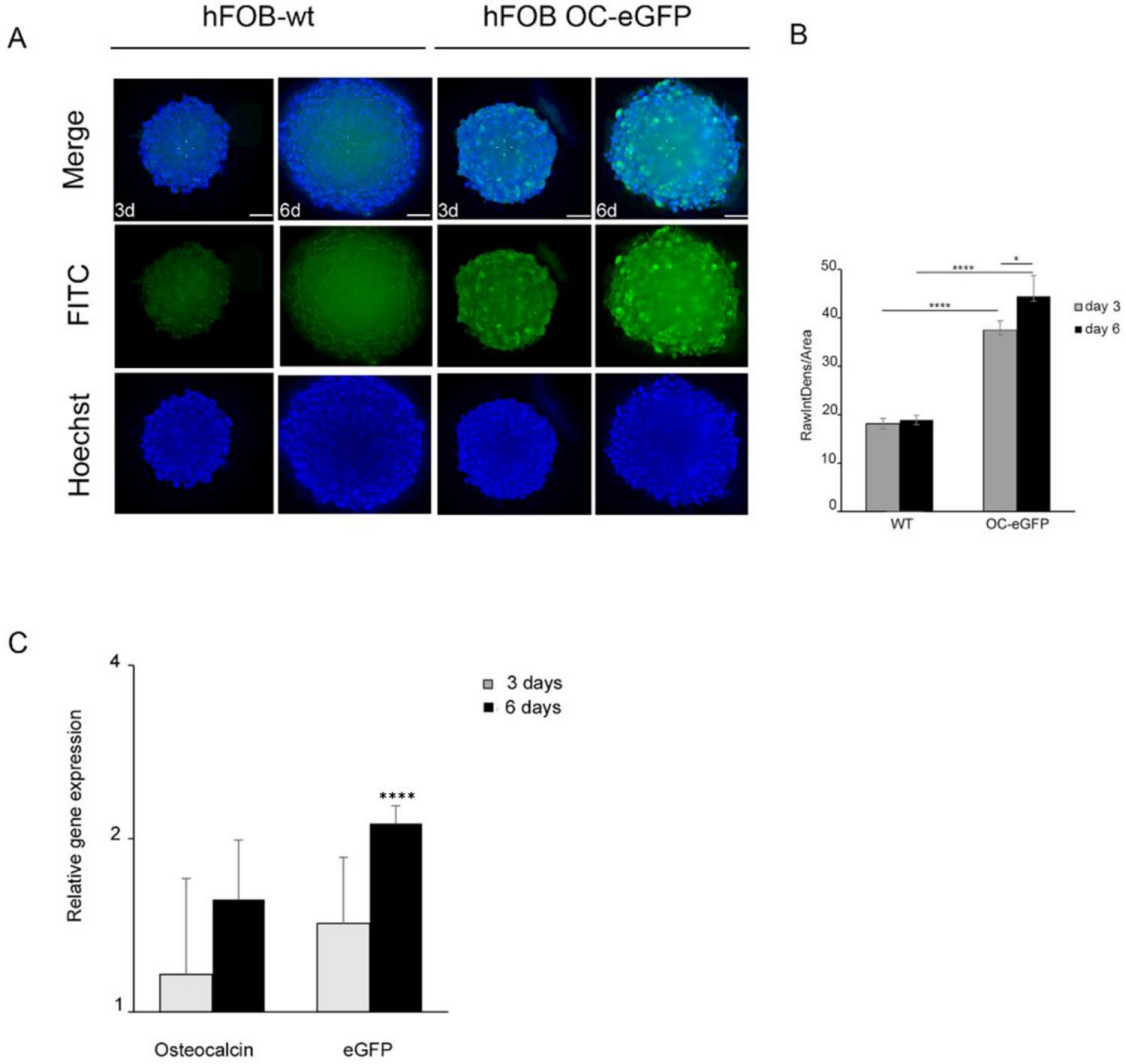
Osteocalcin expression in spheroids under proliferative conditions. Cells expressing eGFP under the control of human osteocalcin promoter (hFOB hOC_eGFP), were used to form spheroids. Displayed are representative, deconvoluted images of spheroids cultivated for 3 and 6 days at different culture conditions at 34 °C. (**A**) eGFP expression in hFOB hOC_eGFP spheroids in growth medium was compared to hFOB wild-type spheroids (autofluorescence). (**B**) Reporter activity of hFOB OC_eGFP spheroids under adipogenic medium was significantly downregulated when compared to osteogenic medium. A semi-quantitative analysis of osteocalcin promotor activity was referred to expression in 2D sub-confluent cultures shortly before spheroid formation. Nuclei were counterstained with Hoechst. For image deconvolution, z-stacks of 40 µm spheroid central-sections were processed with Huygens Essential Software. eGFP intensity estimation was performed by means of ImageJ software on deconvoluted sections. GFP-intensity was normalized to the estimated volume of the spheroids and compared to the value of 1-day-old spheroids. Statistical significance was evaluated by a two-tailed unpaired Student T-test of at least three experiments in triplicate. *P < 0.05; ***P < 0.001; ****P < 0.0001 (**C**) Gene expression analysis of eGFP and osteogenic marker, osteocalcin. Relative expression was based on gene expression of the 2D sub-confluent cultures used to produce spheroids. Two-tailed unpaired Student T-test were applied for determination of statistical significance ****P < 0.0001.

hFOB are preosteoblasts according to analysis, yet as shown here for 3D cultivation, they readily self-propagate structured matrix products in conjunction with calcium deposition. Therefore, we next investigated whether a 3D-cultivated hFOB aggregate not only commences with self-organized, spatially regulated osteogenesis but also continues to differentiate and eventually may further mature to term. For this purpose, an hFOB reporter cell line was used; this cell line, hFOB hOC_eGFP, was derived from hFOB.1.19 and expresses eGFP under the control of the full-length human osteocalcin promoter^15^. Osteocalcin is secreted by osteoblasts during osteoid-matrix production, thereby fostering mineralization during bone formation. Reporter activity was monitored under 3D and proliferative conditions (Fig. 4A). The eGFP fluorescence signal increased over time in culture, while the signal from the wild type hFOB 1.19 spheroids remained unchanged (Fig. 4B). In parallel, we analyzed the mRNA expression of endogenous osteocalcin and eGFP to further evaluate the reporter activity. Normalization was based on undifferentiated 2D-cultivated cells (subconfluent reporter cultures in growth medium from which spheroids had been derived). Both eGFP and endogenous osteocalcin transcripts were upregulated after spheroid formation (Fig. 4C).

**Figure 5:**
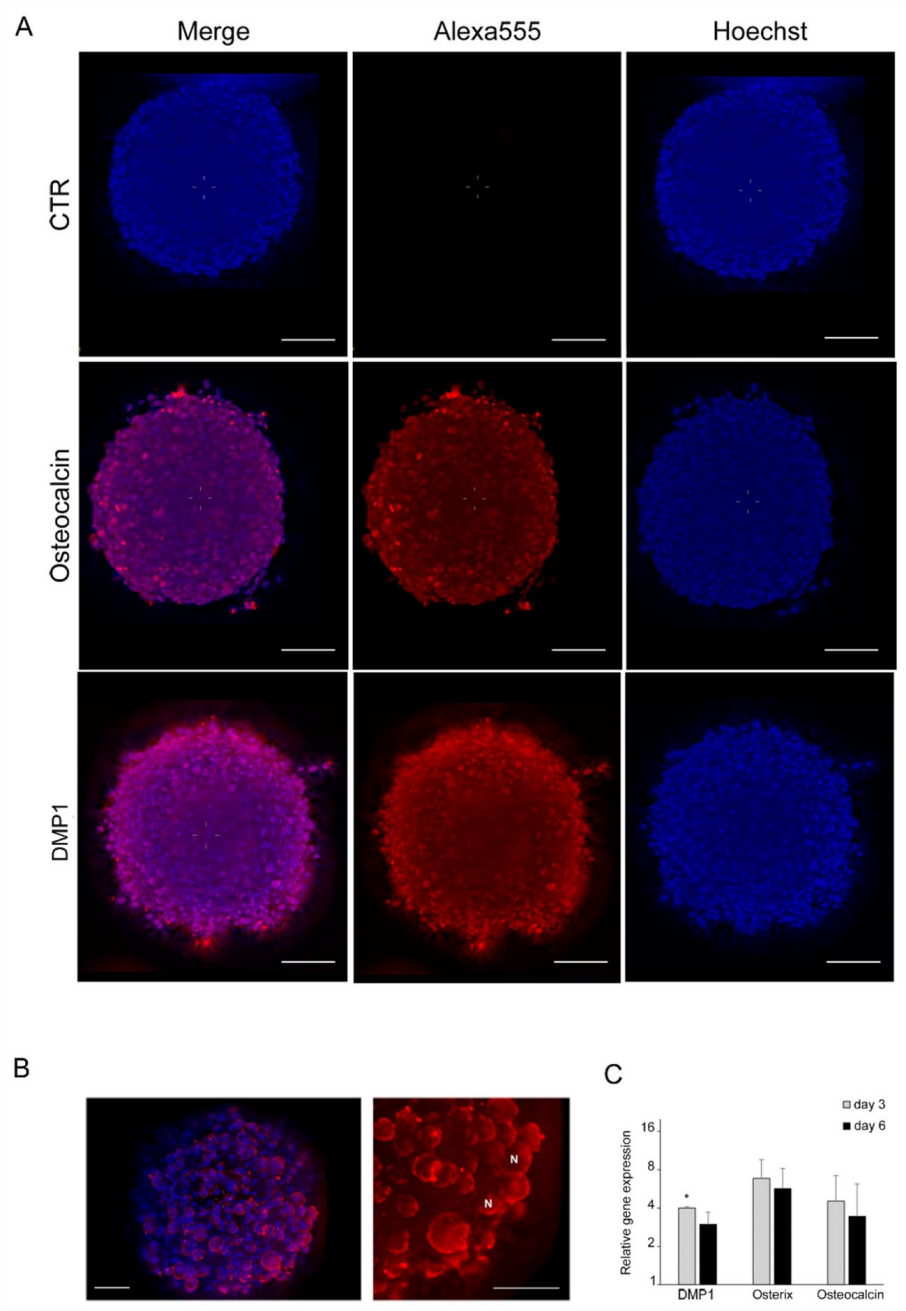
Osteoblast-osteocytes transition markers. Detection of markers indicative for osteocyte differentiation in spheroids formed under proliferative culture condition. (**A**) Deconvoluted images of Osteocalcin and DMP1 (Alexa555 in red) together with nuclear counterstaining with Hoechst 33342 (blue). Before image acquisition, spheroids were cleared with 88% glycerol. Control spheroids were assessed with secondary antibody only. Scale bar, 50 µm (**B**) Expression of DMP1 at higher magnification. DMP1 expression showed a cytoplasmatic localization. The signal is particularly strong in proximity to the plasma membrane, which indicates nascent DMP1 being secreted as component of the osteoid matrix (**C**) Gene expression analysis of DMP1, Osterix and Osteocalcin in 3D cultures in growth medium. Relative expression for each gene was assessed as fold-induction based on the expression of the corresponding gene in 2D sub-confluent cultures used to produce spheroids. Significance was determined by comparing baseline expression of each gene in 2D with the corresponding expression in spheroids. Two-tailed unpaired Student T-test were applied for statistical analysis. *P < 0.05.

Taken together, these findings indicated that the cultivation of hFOB in 3D was sufficient to promote osteogenic differentiation, and further, it could be assumed that differentiation further continued to occur in osteocytes. Hence, the expression of dentin matrix acidic phosphoprotein 1 (DMP1), which is involved in bone mineralization, and is a known marker of preosteocytes^16^, was assessed (Fig. 5). Deconvolution epifluorescence microcopy at the central regions of the 6-day spheroids (Fig. 5A) revealed that the anti-DMP1 antibody bound to cytoplasmic determinants, likely indicating subcellular regions of the secretory pathway. The cytoplasm of the spheroid-derived outgrowth cells was conserved for DMP1 (Fig. 5B). This observation was corroborated by quantitative transcriptional analysis. Increased levels of DMP1 transcripts were accompanied by upregulated Osterix and osteocalcin expression (Fig. 5C). This finding supported the notion that after a few days of cultivation in 3D culture, osteogenic lineage cells are likely prone to mature into osteocyte-like cells.

### Use of hFOB spheroids as a platform for bone marrow cell culture

**Figure 6:**
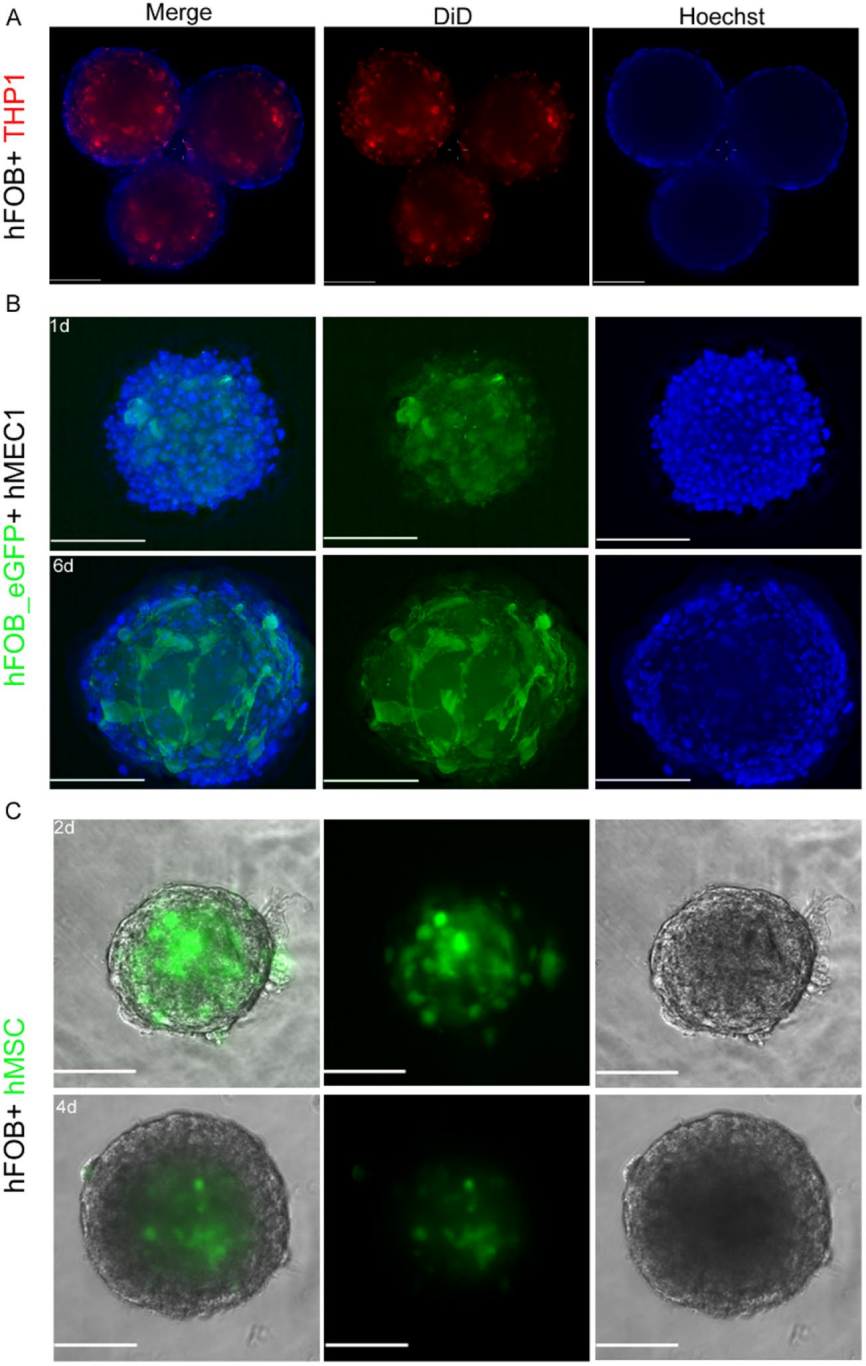
hFOB aggregates constitute a living substrate and 3D-scaffold for different bone marrow cell types. Various cell types were presented to hFOB spheroids in 3D co-culture and could be maintained in hFOB growth medium at 34 °C. Scale bars indicate 100 µm. (**A**) Leukemia monocytes THP1 were labelled with the lipophilic fluorescent dye DiD (red) and added to 3-days old hFOB aggregates. Cultures were kept in hFOB growth medium for 12 days. Deconvolution microscopy revealed the monocytes being present in spheroid cores. Nuclei were counterstained with Hoechst (blue). (**B**) Human microvascular endothelial cells (hMEC1) and hFOB expressing eGFP (hFOB_eGFP; green fluorescence) were mixed at a ratio of 3:1 for spheroid formation. Images were taken one (1d) and six (6d) days after joint spheroid formation with the endothelial cells covering the surface of the aggregate. Nuclei were counterstained with Hoechst (blue) (**C**) Also, wild type hFOB together with human mesenchymal stem cells (hMSC), in the presented case pre-stained with CFSE (green fluorescence), jointly formed stable aggregates. MSCs were found to be located within the spheroid core. Joint aggregates were imaged in bright field illumination; MSC were localized by means of epifluorescence.

To recapitulate cell interactions occurring in bone tissue, we further assessed whether hFOB aggregates can serve as substrates for other cell types that are present in the bone marrow. Peripheral blood monocytes are precursor cells for osteoclasts and play essential roles in osteogenesis and bone remodeling. For these reasons, THP-1 cells, which are human leukemia monocytic cells that can differentiate in vitro into functional osteoclasts were applied^17^. DiD-labeled THP1 cells were added to 3-day-old hFOB spheroids suspended in growth medium. After 12 days, deconvolution microscopy showed DiD-positive cells inside the spheroids. Monocytes appeared to aggregate in highly fluorescent clusters at the periphery of the core (Fig. 6A).

Vascularization of 3D biomolecules is essential in the context of the bone tissue microenvironment and thus relevant when attempting to grow artificial bone-like tissue. We therefore aggregated hFOB_eGFP together with human microvascular endothelial cells (HMEC-1 cells) at a ratio of 3:1. hFOB/hMEC1 spheroids formed rapidly and could be maintained in hFOB growth medium until 6 days after formation. One day after formation, hFOB_eGFP sorted into the innermost aggregates. At later time points, hMEC1 were still present, although they were present mainly in the outer layers. However, the morphology of the hFOB-eGFP cells clearly changed. The aggregates increased in size over time and exhibited a less compact phenotype than did the homogenous hFOB spheroids (Fig. 6B).

Additionally, human stromal cells (hMSCs) were co-cultured with hFOB cells. Two days after hMSC aggregation, cells were labeled with the green-fluorescent agent CFSE and were found in the core of the aggregates. During the next two days, as the hFOB/hMSC aggregates grew, the CFSE level within the core region decreased (Fig. 6C).

## Discussion

Preosteoblasts hFOB1.19, which were originally derived from fetal human bone^18,19^, formed stable cell aggregates in vitro that could be maintained in culture for up to one month with no apparent features of central necrosis. In strong contrast, the inner cell masses of tumor spheroids develop differently. It can form necrotic cores, a clear distinction from vital tissues^20^. Conversely, embryonic cells continue to grow after aggregation without even displaying the onset of necrosis^21–23^. Evidently, cellular viability and extracellular integrity are key in building functional microtissues in vitro.

After the aggregation of approximately 1000 human fetal preosteoblasts, a stable spheroid formed overnight. When such spheroids were cultivated under proliferative conditions for 6 days, the size of the aggregates increased, osteocalcin (OC) expression increased, and extracellular matrix (ECM) was produced. The non-mineralized bone matrix, known as the osteoid, is formed in conjunction with several proteins secreted by osteoblasts. The most abundant non-collagenous component of the osteoid is OC, which binds hydroxyapatite with high affinity and is considered an indicator of bone quality and strength^24^. The expression of osteocalcin is considered a specific marker of mature osteoblasts. In parallel, extracellular calcium appeared within the core regions. Conclusively, in contrast to 2D cultures as previously reported^15^, hFOB aggregates self-reliantly commence with osteogenic differentiation even under proliferative conditions.

Osteogenic development can be both enhanced and suppressed by extrinsic cues. Commonly, osteogenic differentiation in 2D cultures is induced by means of culture media supplemented with dexamethasone, ß-glycerophosphate, or ascorbic acid^25^. hFOB1.19 differentiation can be induced either by cultivating the cells at an unphysiologically high temperature of 39 °C or by supplementing the growth medium solely with ascorbic acid^18^. In our experience, even if a temperature of 39 °C induces rapid differentiation, this temperature is not suitable for long-term culture. In fact, it not only drives high cell death but also has physiological drawbacks linked to the heat stress response. With the objective of providing a better research tool, we therefore adopted a temperature of 34 °C in our experiments. To enforce osteogenesis in 3D cultures, hFOB spheroids were cultured in the presence of differentiation factors in a manner comparable to that used for conventional 2D protocols. Relatively compact spheroids developed when aggregates were stimulated with osteogenic inducers. The growth of the aggregates ceased, the rate of apoptosis increased, the levels of extracellular calcium and matrix production increased, and the expression of the mineralizing osteocyte marker DMP1 increased. Apoptosis opens spaces to promote bone growth during embryonic and fetal development. Hence, mineralization is accompanied by apoptosis^26,27^, which is also a characteristic aspect of tissue self-renewal^28,29^. In bone, approximately 60–80% of osteoblasts undergo apoptosis after mineralization, while residual osteoblasts differentiate into osteocytes or into bone lining cells^30^. During osteogenic treatment, hFOB1.19 produces apatite and other types of minerals^31^. Whether hydroxyapatite-like mineralization occurs in the 3D model presented here can be further confirmed by focused compositional and structural analysis, including TEM–EDX^32^. According to the fluorescence data of calcium deposition, osteogenic induction medium promoted faster and more substantial differentiation than growth medium. Nevertheless, mineralization and increased expression of the osteocytic marker were not supported by abundant osteoid production or osteoblast proliferation, indicating that the balance between osteoblasts and osteocytes was affected and that the cells were polarized toward mineralizing DMP1^+^ osteocytes. To establish an organoid-like model, the differentiation processes must be balanced since in vivo bone homeostasis is achieved by the control that osteoblasts and osteocytes exert on each other and on other cell types, such as osteoclasts. Based on this observation, we concluded that medium lacking conventional supplements such as hormones or growth factors is sufficient to render aggregates apt to self-organize in a way that promotes self-reliant differentiation of hFOB specifically towards an osteocytic lineage. Indeed, under this same culture conditions adipogenic differentiation could not be observed. On the contrary, osteoblastic markers, such as osteocalcin and Osterix, are upregulated in hFOB spheroids under proliferative conditions, and the latter is a key requirement for osteoblast differentiation and bone mineralization^33,34^. The mineralization-associated gene DMP1 was also strongly upregulated, as well as Podoplanin, an established marker for osteocytes^35^. Eventually, DMP1 is secreted into the extracellular matrix, where it coordinates the nucleation of hydroxyapatite^36^. Sclerostin, a glycoprotein produced mainly by mature osteocytes, could not be detected in hFOB aggregates. If not for technical reasons, this could indicate that the chosen 3D culture conditions are still insufficient to promote terminal differentiation of osteocytes^37^.

This finding calls for creative measures that improve and enhance the functional potential of hFOB spheroids. One strategy is to optimize hFOB self-organizing aggregates by incorporating other cell types that generate bone, such as osteoclasts and endothelial and stromal cells. To date, the technical constraints to accomplish this goal are not well understood. The prime aspect with respect to cellular well-being is a common medium that promotes nonselective cell survival, amble growth and decisive cellular interaction of all cell types incorporated into the microtissue. For this reason, we evaluated the influence of hFOB growth medium in combination with other cell types on spheroid formation and stability. First, human monocytes (THP1) were added to preformed spheroids under hFOB culture conditions in complete DMEM:F12 medium at a temperature of 34 °C. Notably, these changes resulted in the migration of monocytes into spheroid core regions. After 12 days of culture, monocytes labeled with a fluorescent membrane dye could be distinguished and appeared to cluster together, as previously described in other culture models^38,39^. As vascularization plays a decisive role in establishing physiologically relevant microtissues in vitro^40^, we next incorporated microvascular endothelial cells (hMECs) into spheroids by combined cell aggregation. Additionally, these aggregates grew and increased in size after 6 days of culture. Next, we applied bone marrow stromal cells, which are important determinants of bone remodeling/homeostasis and hematopoiesis^41^. Therefore, human mesenchymal stem cells (hMSC) were first labeled with the cytoplasmic dye CFSE and subsequently co-cultured with hFOB to form compact spheroids. MSC localized to the central regions of the aggregates; these cells increased in size and remained compact for 4 days in hFOB culture conditions. Fluorescence in the MSC ceased over time but remained clearly detectable, which led us to speculate that MSC may also proliferate under these conditions. These preliminary results demonstrate the growing potential of multicellular spheroids for future research addressing assays amenable to address complex tissue function.

Taken together, our results indicate that osteoblasts that construct spheroids activate pathways, which are likely activated during osteogenic differentiation in vivo. Although the 3D-model presented here is incapable of growing mature osseous tissue with sturdy mineralized biomaterial, the complexity of developmental pathways driving early osteogenic differentiation is most likely established and displayed. Presumably attributable to their fetal origin and not unexpected, hFOB1.19 unveil also somatic cellular characteristics rather than behaving exclusively like an immortal line^15^. Provided these special features, they resemble primary human osteoblasts more closely than for instance transformed cell lines, such as SAOS2. The here presented approach could thus be considered an improved way of studying cell–cell interactions, molecular signals, or physical influences that affect osteogenesis ex vivo. We therefore suggest 3D-cultivation of hFOB1.19 being a superior alternative to primary osteoblast cultures. Primary human osteoblasts are not only hard to acquire, yet they are due to donor-variations, such as sex, age and overall bodily constitution, seemingly hard to compare to each other. A particular technical constraint of hFOB1.19 could be that optimal cultivation appears to take place at 34 °C. We however experienced that this set temperature could support also the viability of other cell types when co-cultured with hFOB. Hence, the fact that many cell types exhibit thermal plasticity and undergo putative, yet not fully understood changes, may result in deceptive interpretation of experimental observations^42,43^

Hence, this surrogate tissue model appears apt for further development by combining even more cell types together and thus, provides a novel tool for investigations of bone cell biology and pathology^44,45^.

## Methods

### Cell culture

The immortalized human fetal osteoblast cell line hFOB1.19 (hFOB1.19) was purchased (ATCC® CRL-11372™). It was cultured according to the protocol specified by the repository and regularly tested for mycoplasma contamination. hFOB1.19 cells were maintained in growth medium (GM): DMEM:Ham’s F12 (Sigma, D6421) supplemented with 10% fetal bovine serum (FBS), 2.5 mM L-glutamine, 100 U/mL penicillin and 100 μg/mL streptomycin, 300 µg/ml G418 (Sigma, G8168). All the experiments were carried out at 34 °C and 5% CO_2_. Two monoclonal hFOB1.19 cell lines expressing enhanced green fluorescent protein (eGFP) under the control of the i) human full-length osteocalcin promoter (hFOB hOC_eGFP) and ii) the human ubiquitinase (UbC) promoter (hFOB_eGFP) were generated previously by lentivirus transduction^15^. Cultivation was conducted at 34 °C in growth media. Osteogenesis was triggered in spheroids by incubating the cells at 34 °C in osteogenic medium (OM): DMEM:Ham’s F12 mixture containing 2% FBS, 500 µM ascorbic acid and 10 nM dexamethasone (Sigma, D4902). For adipogenic differentiation, the cells were incubated in adipogenic medium (AM) supplemented with 0.5 mM isobutyl‐methylxanthine (Sigma, I5879), 1 μM dexamethasone, 10 μM insulin (Sigma, l9278), and 60 μM indomethacin (Sigma, I7378). Spheroids were formed by incubating 10^3^ cells in ultralow attachment U-bottom 96-well plates (Greiner)^46^. Further cultivation was carried out in 96-well-plates at 34 °C and 5% CO_2_, with medium changes performed every second day. eGFP fluorescence was quantified by summing all pixel values in the region of interest (raw integrated density) by means of image analysis (ImageJ, Fiji open source) at 3 and 6 days post-aggregation. eGFP signals related to growth and osteo- and adipogenic media were normalized to the area, and differences were statistically evaluated via unpaired t tests.

THP-1 cells were purchased (ACC16; DMSZ Leibniz-Institut-Deutsche Sammlung für Mikroorganismen und Zellkulturen GmbH). They were expanded in RPMI 1640 medium supplemented with 5% FBS, 100 U/mL penicillin and 100 μg/mL streptomycin. Human mesenchymal stem cells-hMSCs (Cambrex, East Rutherford, NJ) were expanded in α-MEM supplemented with 20% FBS, 100 U/mL penicillin and 100 μg/mL streptomycin. Both THP1 cells and hMSCs were cultivated as monocultures at 37 °C. For all co-culture experiments, complete DMEM:Ham’s F12 growth medium was used.

### Immune and histochemical staining

The spheroids were fixed in 4% paraformaldehyde in PBS for 30 min and permeabilized with 0.3% Triton-X-100 solution in PBS for 15 min at room temperature. To prevent nonspecific antibody binding, the specimens were incubated for 1 h at room temperature in blocking solution (0.2% Triton X-100, 0.05% Tween-20, 3% bovine serum albumin in PBS) with gentle shaking. After blocking, the spheroids were incubated with primary antibodies overnight (ON) at 37 °C with gentle shaking. The primary antibodies used were as follows: anti-human BGLAP (ABclonal #A6205, Lot 1152580201; anti-human DMP1 #A16832, Lot 5500009628). The antibodies were diluted 1:100 in antibody buffer (1% bovine serum albumin, 0.2% Triton X-100, 0.05% Tween-20 in PBS). After washing, the samples were stained with the corresponding secondary antibody and nuclear dyes (Hoechst 33342, Sigma #14533) for 4 h at 37 °C with gentle shaking. The secondary antibodies used were as follows: anti-rabbit AlexaFluor 555 (Cell Signaling #4413, Lot 10) and anti-mouse AlexaFluor 555 (Cell Signaling #4409, Lot 11). The secondary antibodies were diluted 1:800 in antibody buffer together with Hoechst 33342 (10 µg/ml). After washing, the spheroids were cleared with glycerol as previously described^47^.

Calcium deposition was detected using Xylenol Orange (XO), Calcein blue and Von Kossa. Outgrown cultures were first stained with the fluorescence dye Calcein blue according to Yu-Hsiung Wang and colleagues^11^. After image acquisition, cultures were fixed with 4% paraformaldehyde for 30 min, and susequently stained with 5% Silver Nitrate solution. After 60 min exposure to ultraviolet light, cultures were rinsed in distilled water, incubated with 2.5% Sodium Thiosulphate for 5 min and washed in distilled water.

Calcium staining of living cells was carried out by incubating specimens overnight with 20 µM XO (Fluka, #33825). After extensive washing, the samples were resuspended in phenol red-free medium.

Hoechst 33342 (10 µg/ml) and propidium iodide (1 µg/ml) were applied to the growth media for 45 min at 37 °C. After staining, the spheroids were washed with PBS to remove residual dye and transferred to phenol-free medium.

### Deconvolution microscopy

Image stacks from the central region of the spheroids were acquired by means of widefield microscopy (Leica DMi8 controlled by LAS X software 3.4.2, Leica Microsystems). Image deconvolution was performed using Huygens Essential Software (Scientific Volume Imaging, Hilversum, The Netherlands). Image stacks of 30–40 µm in the center of the micromass were acquired by automatically optimized spacing. Only images with a Nyquist number ≥ 0.9 were used for further analysis. Deconvolution was performed by applying the following settings: (i) automatically calculated point-of-spread-function (PSF), (ii) logarithmic function for the image intensity histogram, and (iii) classical maximum likelihood estimation (CMLE) as a deconvolution algorithm. Additional parameters were calculated from the imaging conditions according to the software instructions (Table 1).

**Table 1:** Deconvolution parameters.

Refractive index of specimen medium	1.338 (water) 1.42–1.46 (Everbrite hard set mounting medium)
Background estimation	Widefield
Area radius	0.2–0.5 µm
Maximum iterations	50–80
Signal to noise ratio (SNR)	40–50
Quality threshold	0.01
Iteration mode	Optimized
Bleaching correction	If possible
Brick layout	Auto

### Histology

The process of spheroid collection, paraffin embedding, and staining was performed according to the methods of Clayton et al.^48^ The aggregates were transferred to a 15 ml tube and centrifuged at 300×*g* for 2 min. The supernatant was discarded, and the sedimented spheroids were washed twice with PBS and then fixed with 10% neutral-buffered formalin for 10 min at room temperature. The fixative was removed and replaced with PBS by washing the pellet twice. Specimens were decalcified by applying decalcifier soft solution (Roth, #6484.1) according to the manufacturer’s recommendations. Thereafter, the samples were rinsed three times in PBS and transferred to a small tube. Then, 1% agarose solution (Agarose Type I-A Low EEO, Sigma, A-0169) was added, followed by centrifugation and casting for 1 h at room temperature. The spheroid pellet was then gently removed and wrapped in Bio-Wrap (Surgipath Bio-Wrap, VWR #720-2430) before being transferred into an embedding cassette. Ethanol infiltration started with 70% and 80% ethanol for 10 min, followed by 95% ethanol for 15 min, three changes of 100% ethanol for 10 min, and three changes of xylene for 10 min. Samples were transferred into paraffin for 1 h with two changes of fresh paraffin for 15 min each. Sections were cut at 5 µm, mounted onto a hydrophilic glass slide (Superfrost Ultra Plus, Menzel #1014356190) and stained with hematoxylin and eosin (H&E, Thermo Scientific #411160250). When preparing for fluorescence immunohistochemistry, sections were first incubated in preheated citrate buffer (10 mM, 0.05% Tween, pH 6) at 90 °C. The slides were cooled to RT in citrate buffer for 15 min, rinsed with TBS-Tween (0.05% Tween), permeabilized in 1% BSA and 0.4% Triton X-100 in PBS for 20 min and incubated with blocking solution containing 3% BSA, 0.2% Tween-20 and 0.2% Triton X-100 in PBS for 1 h at RT. The blocking solution was removed, and the sections were incubated with anti-Lamin A/C (Santa Cruz Biotechnology, sc-376248, Lot H2719), mouse IgG isotypes (BioLegend #400101, Clone: MOPC-21, Lot B142616), or 1 × PBS as a negative control at 4 °C overnight in a humidified chamber, followed by incubation with secondary antibodies (anti-mouse AlexaFluor 555, 1:500) for 1 h at RT. Sections were washed and counterstained with 0.2 µg/ml DAPI (Sigma, # D9542).

### Apoptosis analysis

The spheroids were fixed with 4% paraformaldehyde for 30 min and decalcified with CUBIC-B according to the manufacturer’s recommendation (TCI, #T3780). After permeabilization with 0.3% Triton-X100 in 0.1% sodium citrate (pH 6) for 30 min, the spheroids were incubated for 1 h in blocking solution (3% bovine serum albumin in PBS). TUNEL staining was carried out according to the manufacturer’s instructions (In Situ Cell Death Reaction Kit, Roche, #1684817), with the sole exception that the incubation was performed overnight at room temperature. Hoechst 33342 (10 µg/ml) was used as a nuclear counterstain.

To inhibit apoptosis, the aggregates were cultured in GM supplemented with 50 µM pancaspase inhibitor (Z-VAD-FMK, Targetmol Cat# T6013). Five days posttreatment, the spheres were stained with propidium iodide (PI) or Xylenol Orange, and the nuclei were counterstained with Hoechst 33342.

### Quantitative PCR

Total RNA was isolated from 100 spheroids with a Direct-zol RNA MicroPrep Kit (Zymo Research, R2050), subjected to DNAse treatment, quantified with a NanoDrop 1000 spectrophotometer (Thermo Fisher Scientific) and stored at − 80 °C. Complementary DNA (cDNA) was synthesized from 100 ng of total RNA using the LunaScript^®^ RT SuperMix Kit (New England Biolabs, E3010L). Only RNA samples with an A_260_/_A280_ ratio ≥ 1.9 were used, and reverse transcription was carried out in duplicate. Quantitative PCR was performed on an AriaMX Real-Time PCR System (Agilent) with Luna Universal qPCR Master Mix (New England Biolabs, M3003L; SYBR channel) using 2 µl of prior diluted cDNA (1:5). Non-template controls (NTCs) were included on each plate. To determine primer specificity, melting curves were generated after 40 cycles of PCR. The fold difference in gene expression was calculated using the ΔΔCt method, and normalization was performed according to the expression of the reference gene *YWHAZ*. The primers used spanned exon junctions to avoid genomic DNA amplification; the sequences are given in Table 2. Statistical significance was evaluated by a two-tailed unpaired Student’s t test for at least three experiments performed in triplicate.

**Table 2:** Primers for qPCR.

Gene target	Primer (5′ – 3′)	Annealing Temp (°C)	Amplicon Length	Accession Number
Osteocalcin	F: AAGAGACCCAGGCGCTACCT	61.4	110	NM_199173.6
	R: AACTCGTCACAGTCCGGATTG	59.8		
eGFP	F: AAGTTCATCTGCACCAC	57.3	115	MK387175.1
	R: AAGTCGTGCTGCTTCATGTG	57.3		
Osterix	F: CCTGCTTGAGGAGGAAGTTCA	59.8	83	NM_152860.2
	R: GGCTAGAGCCACCAAATTTGC	59.8		
ALP	F: CTTCAAACCGAGATACAAGC	55.3	125	NM_000478.6
	R: TCAGCTCGTACTGCATGTC	56.7		
DMP1	F: GAGCAGTGAGTCATCAGAAGGC	62.1	138	NM_004407.4
	R: GAGAAGCCACCAGCTAGCCTAT	62.1		
YWHAZ	F: AAGTTCTTGATCCCCAATGCTT	56.5	196	NM_003406.4
	R: GTCTGATAGGATGTGTTGGTTGC	60.6		

#### Statistical analysis

The data are shown as the mean ± s.d.; n is indicated in the figure legends. Statistical analyses were performed using an unpaired two-tailed Student's t test. A P value of less than 0.05 was considered to indicate statistical significance.

### Supplementary Information

Supplementary Figures.

## Data Availability

The datasets used and/or analyzed during the current study available from the corresponding author on reasonable request.
