# Supplementary material for: Aggregation of human osteoblasts unlocks self-reliant differentiation and constitutes a microenvironment for 3D-co-cultivation with other bone marrow cells
Source: Sci Rep. 2024 May 6;14:10345. doi: 10.1038/s41598-024-60986-8 (PMC11074281; doi:10.1038/s41598-024-60986-8)
Supplement: Supplementary file 1 — Supplementary Figures. [file 41598_2024_60986_MOESM1_ESM.docx]

**Supplemental Material**

**Supplemental Figure 1 (Fig. S1)**

**
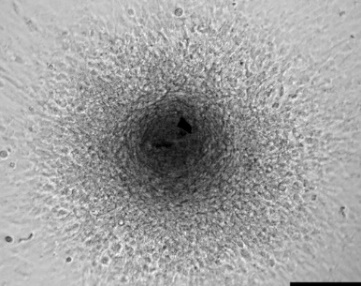

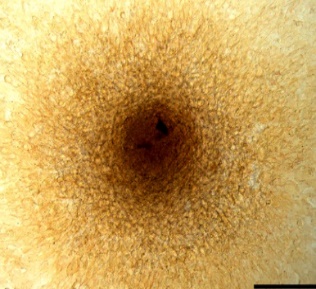

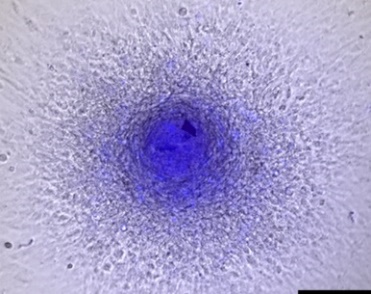
A** Calcein Blue Von Kossa Bright Field

**B** Podoplanin (E11) Hoechst merge


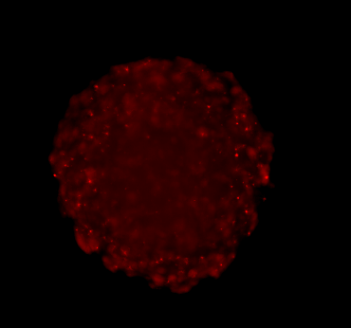

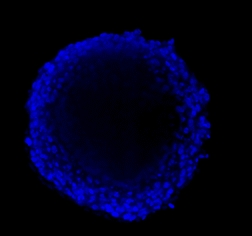

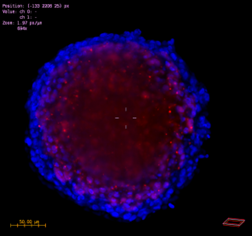


**Fig.S1** **Osteogenic differentiation in hFOB osteoblasts aggregates**

A) Representative 6-day-old spheroid in growth medium were allowed to attach and spread into a tissue cultures plate. Extracellular calcium deposits were visualized by Calcein Blue (live staining) followed by Von Kossa. Signals for both dyes localized in the middle of the culture, which corresponds to the core of the spheroid. Scale bar 100µm.

B) Deconvoluted image of expression of an osteocytic marker Podoplanin (Alexa555 in red) together with nuclear counter-staining with Hoechst 33342 (blue) in a representative 6-day-old spheroid in growth medium. Before image acquisition, spheroids were cleared with 88% glycerol. Scale bar, 50 µm

**Supplemental Figure 2 (Fig. S2)**

**

A B
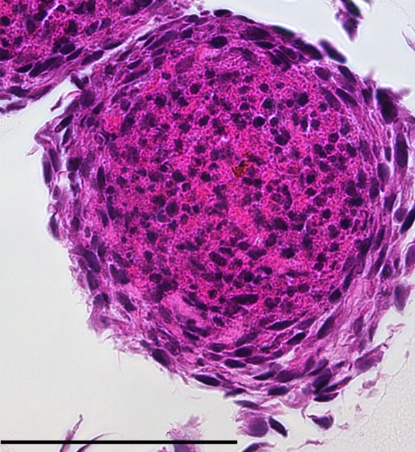
**

**Fig.S2** **Adipogenic differentiation in hFOB osteoblasts aggregates**

A) Representative H&E histological images of hFOB cell aggregates grown under proliferative condition (growth medium) over 6 days. Paraffin-embedded spheroids slices were stained with H&E and did not show fat vacuoles usually seen in adipogenic differentiation.

B) Gene expression analysis of adipogenic, PPARg2. Relative expression of PPARg2 gene in spheroids grown for 6 days in growth medium (GM) or adipogenic medium (AM). Fold induction calculation was based on gene expression of the 2D sub-confluent cultures in growth medium. Two tailed unpaired Student T-test was applied for determination of statistical significance. **P<0,001; ns= not significant.

**Supplemental Figure 3 (Fig. S3)**


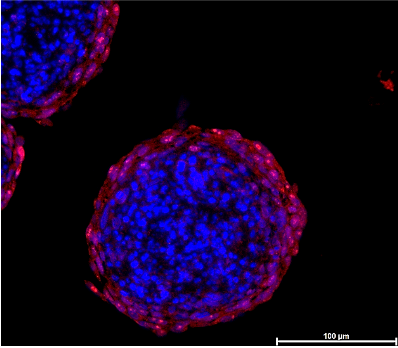

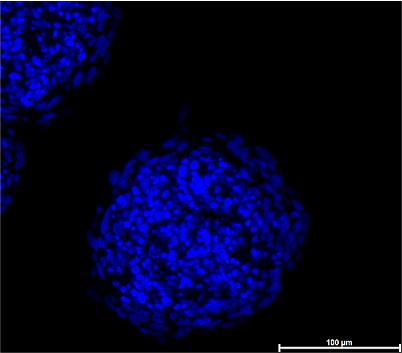

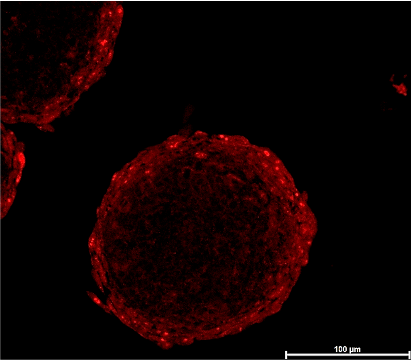
Ki-67 DAPI Merge

**Fig.S3** **proliferation of hFOB osteoblasts aggregates**

Deconvoluted image of the expression of the proliferation marker Ki-67 (in red) together with nuclear counter-stain DAPI (blue) in a representative spheroid in growth medium. Before image acquisition, spheroids were cleared with 88% glycerol. Scale bar, 100 µm
